# HPLC Separation of 2-Ethyl-5(6)-methylpyrazine and Its Electroantennogram and Alarm Activities on Fire Ants (*Solenopsis invicta* Buren)

**DOI:** 10.3390/molecules23071661

**Published:** 2018-07-07

**Authors:** Ya-Ya Li, Yong-Yue Lu, Min Lu, Hong-Yi Wei, Li Chen

**Affiliations:** 1 State Key Laboratory of Integrated Management of Pest Insects and Rodents, Institute of Zoology, Chinese Academy of Sciences, Beijing 100101, China; liyaya0107@163.com (Y.-Y.L.); lumin@ioz.ac.cn (M.L.); 2 Department of Plant Protection, College of Agronomy, Jiangxi Agricultural University, Nanchang 330045, China; 3 Red Imported Fire Ant Research Centre, South China Agricultural University, Guangzhou 510642, China; luyongyue@scau.edu.cn

**Keywords:** 2-ethyl-5-methylpyrazine, 2-ethyl-6-methylpyrazine, regio-isomers, electroantennogram (EAG), alarm pheromone, HPLC

## Abstract

2-Ethyl-3,6-dimethylpyrazine (EDMP) was an alarm pheromone component isolated from the mandibular gland of the red imported fire ant, *Solenopsis invicta* Buren. Several pyrazine analogues have been previously found to elicit significant alarm responses in *S. invicta* workers. This study aimed to separate the commercially available 2-ethyl-5(6)-methylpyrazine (EMP), i.e., a mixture of 2-ethyl-6-methylpyrazine (2E6MP) and 2-ethyl-5-methylpyrazine (2E5MP), and to examine both electroantennogram (EAG) and behavioral responses of *S. invicta* workers to EMP and the purified isomers. HPLC separations were achieved using a polysaccharide chiral stationary phase (Chiralpak AD-H) column with both mobile phases: Cyclohexane/isopropanol, and hexane/isopropanol. A ratio of 99:1 was selected for the separation of EMP at semipreparative level. The structures of the isomers obtained through the cyclohexane/isopropanol mobile phase were confirmed by detailed analyses of 2D-HSQC- and -HMBC-NMR data. The two isomers showed differential methine C–H correlations evidenced by 2D-HMBC-NMR spectra. The two concentrated fractions obtained through hexane/isopropanol mobile phase were subjected to EAG test and behavioral bioassay on *S. invicta* workers. The two HPLC−purified isomers, 2E6MP and 2E5MP, and their mixture (1:1) at same dose elicited similar EAG and alarm responses, indicating that these two isomers are equally active. The 2D-NMR−spectroscopic characterization, and electrophysiological and alarm activities of 2E6MP and 2E5MP were reported here for the first time.

## 1. Introduction

**Figure 1:**
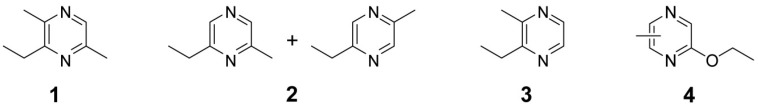
Chemical structures of pyrazines. **1**, 2-ethyl-3,6-dimethylpyrazine (EDMP); **2**, 2-ethyl-5(6)-methylpyrazine ((EMP) (2-ethyl-6-methylpyrazine (2E6MP) + 2-ethyl-5-methylpyrazine (2E5MP))); **3**, 2-ethyl-3-methylpyrazine; **4**, 2-methyl-3(5 or 6)-ethoxypyrazine (MEOP).

**Figure 2:**
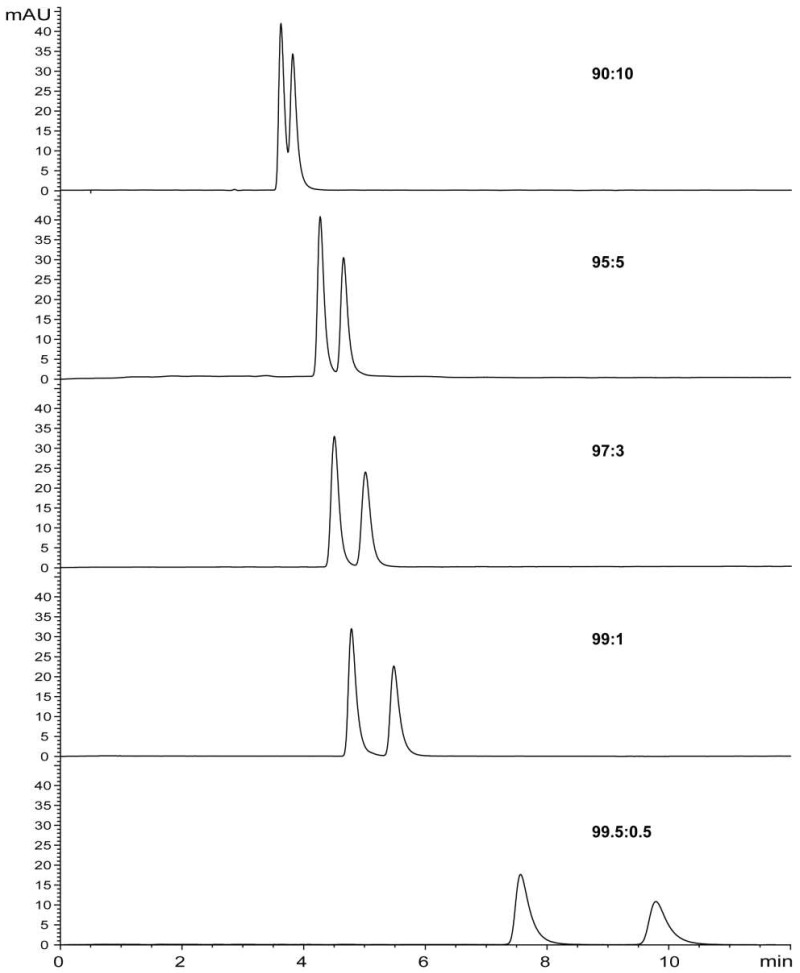
Chromatograms obtained from HPLC analysis of EMP using cyclohexane/isopropanol as eluent.

**Figure 3:**
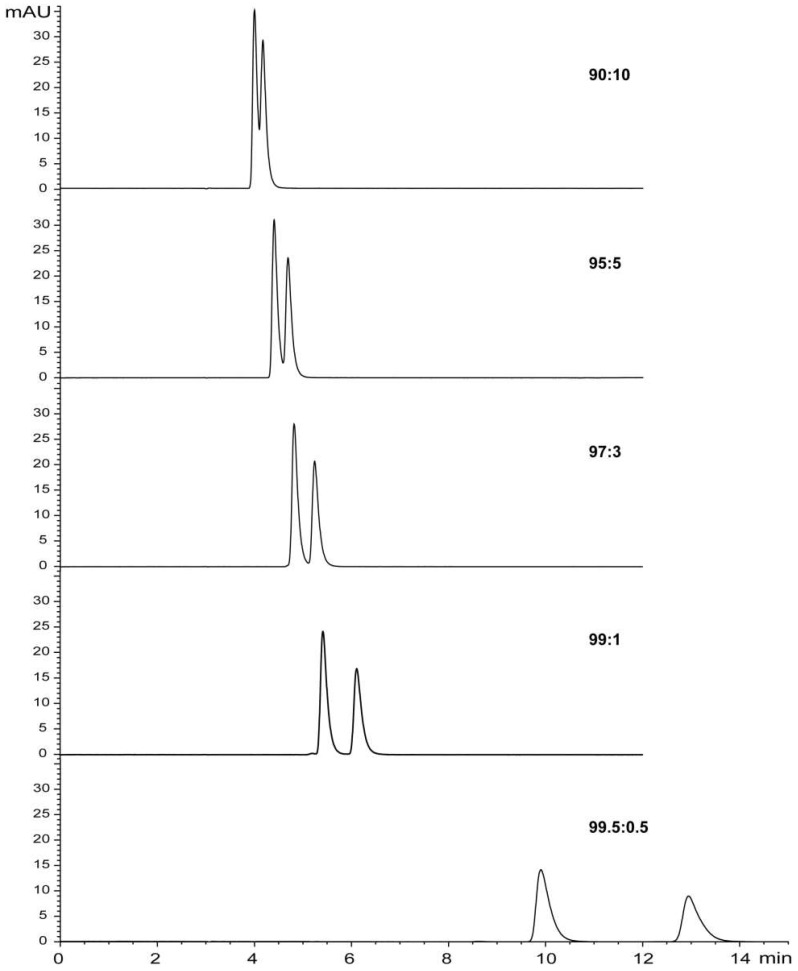
Chromatograms obtained from HPLC analysis of EMP using hexane/isopropanol as eluent.

Pyrazine and its derivatives are one class of the most important flavor compounds responsible for cocoa taste and have been widely used as food flavor additives in the food industry. Pyrazines also contribute substantially to flavor formed in various roasted, toasted and heated foods [1,2,3]. They are generated primarily from a Maillard reaction between amino acids and sugars during toasting [4]. The most abundant alkylpyrazines in roasted coffee were 2-methylpyrazine, 2,6-dimethylpyrazine, 2,5-dimethylpyrazine, 2-ethylpyrazine, 2-ethyl-6-methylpyrazine (2E6MP, Figure 1), 2-ethyl-5-methylpyrazine (2E5MP, Figure 1), and 2,3,5-trimethylpyrazine [5]. These structure-related alkylpyrazines may exhibit similar properties and contribute to the remarkable physiological activity of coffee. Pyrazines disclose a nut-like aroma in many different types of foods thermally processed, and the sensory importance of alkylpyrazines (or alkenylpyrazines) to animals and human being has been highlighted [6,7,8].

Pyrazines have been of interest since many are implicated as trail and alarm pheromones in insects [9]. Many studies have investigated the chemistry and role of pyrazines in ants. Trail following function has been attributed previously to di- and tri-alkylpyrazines in ants. Although 2,5-dimethylpyrazine, 2,3,5-trimethylpyrazine, and 2-ethyl-3,6-dimethylpyrazine (EDMP, Figure 1) have been found in the venom gland of several ant species, only EDMP was active as a trail pheromone in *Atta sexdens rubropilosa* [10], *A. sexdens* [11], and *Manica rubida* [12,13]. In addition to *Atta*, *Manica*, *Pogonomyrmex*, and *Tetramorium* spp., EDMP was also identified as either a major or minor trail pheromone component from the venom gland of several ant species of *Messor*, *Myrmica*, and *Pheidole* [11,14,15,16,17,18]. Trialkyl substituted pyrazines that show 2,5-dimethyl-3-alkyl or 2,6-dimethyl-3-alkyl substitution patterns are known components of alarm pheromones derived from mandibular gland of ant species of Dolichoderinae, Formicinae, Myrmiciane, and Ponerinae [19]. For instance, 2,5-dimethyl-3-isopentylpyrazine released escaping behavior in workers of *Hyponera opacior* and *Ponera pennsylvanica* [20]. 2,5-Dimethyl-3-*n*-propylpyrazine was identified as the sole pyrazine in the Australian bull ant, *Myrmecia gulosa* [21]. 2,5-Dimethyl-3-(3-methylbutyl)pyrazine was identified as the mandibular alarm pheromone of the little fire ant *Wasmannia auropunctata* [22]. The major components in mandibular gland secretions of *Odontomachus troglodytes* and *Brachyponera sennaarensis* were 2,6-dimethyl-3-*n*-butyl- and -*n*-pentylpyrazine [23]. Vander Meer et al. [24] identified EDMP from the mandibular gland of the red imported fire ant, *Solenopsis invicta*, which was the first report of this pyrazine functioning as an alarm pheromone. In previous reports, we have screened alarm activity of analogue series of EDMP, and found that 2,3,5-trimethylpyrazine, 2-ethyl-3,5-dimethylpyrazine, 2,3-diethyl-5-methylpyrazine, 2-methoxy-3-methylpyrazine, 2-ethoxy-3(5 or 6)-methylpyrazine, and 2-chloro-3-methoxypyrazine elicited significant alarm responses in *S. invicta* workers [25,26]. In our continuing efforts to search for more pyrazine analogues with alarm activity, 2-ethyl-5(6)-methylpyrazine (EMP, Figure 1) was found to be as active as commercially available mixture of fire ant alarm pheromone isomers, 2-ethyl-3,5(6)-dimethylpyrazine. As 2-ethyl-6-methylpyrazine (2E6MP) and 2-ethyl-5-methylpyrazine (2E5MP) are not commercially available, separation of EMP is required for testing the activity of individual compounds in commercial mixtures.

Reversed-phase (RP)-HPLC is commonly applied in the separation of alkaloids. Some alkaloids are often successfully separated on RP columns in simple eluent systems with only organic modifier and water. For instance, caffeine was determined in green tea by RP-HPLC in an eluent system containing CH_3_CN and water [27]. Liquid Chromatographic analyses of pyrazines are usually achieved on octadecyl silica (ODS) C18 column with CH_3_CN/water or MeOH/water mobile phase [28,29,30,31,32,33,34]. In our previous report, 2-ethyl-3,5-dimethylpyrazine and EDMP were successfully resolved on C18 column with ≤25% CH_3_CN in water at a flow rate as low as 0.2 mL/min [26]. However, this system was not applicable for separation of 2E6MP and 2E5MP. It is, therefore, very important to develop a new method which allows simple and practical separation of EMP.

We report here the fast separation of EMP with a polysaccharide chiral stationary phase (Chiralpak AD-H) (amylose tris-(3,5-dimethylphenyl-carbamate) column. Two-dimensional (2D) NMR analysis was used to determine the chemical identity of the two HPLC-purified fractions. We also tested electroantennogram (EAG) and behavioral activity of the two HPLC-purified fractions in comparison with that of 1:1 mixture.

## 2. Results and Discussion

### 2.1. Preparative HPLC

Separation of 2E6MP and 2E5MP in the commercial product can be accomplished on Chiralpak AD-H column using cyclohexane/isopropanol or hexane/isopropanol mobile phase. Figure 2 and Figure 3 illustrate elution profiles of EMP at a flow rate of 1 mL/min with variable ratios of cyclohexane/isopropanol and hexane/isopropanol mobile phase, respectively. The injection results in two chromatographic peaks which correspond to the two regio-isomers. The separation efficiency increases with the decrease of isopropanol ratio in mobile phases. At a ratio of 99:1 (*v*/*v*), the two regio-isomers are completely baseline separated. The best discrimination for EMP was obtained with a ratio of 99.5:0.5 (*v*/*v*). Chiralpak AD-H column is often used for the HPLC enantiomeric separation of racemic alkaloids with hexane/isopropanol as the mobile phase [35]. Amylose tris-(3,5-dimethylphenyl-carbamate) is one of the most widely used polysaccharide-based chiral stationary phases, featuring excellent separation capabilities not only for enantiomers but also for difficult resolutions of non-chiral compounds like closely related regio-isomers and diastereomers. This feature stimulated its application for non-chiral separation of EMP and related pyrazines. By using hexane-isopropanol (99.5:0.5) mobile phase, 2-methyl-3(5 or 6)-ethoxypyrazine (MEOP, Figure 1) was readily separated into two peaks on Chiralpak AD-H column (Supplementary Material Figure S1). Gas chromatography–mass spectrometry (GC-MS) analyses on polar column DB-WAX and non-polar column HP-5ms, and ^1^H-NMR analysis all confirmed that there were only two components in the commercial product of MEOP. However, no satisfactory separation of EDMP was achieved on the Chiralpak AD-H column with the above mobile phases.

We used a ratio of 99:1 (*v/v*) for rapid scaling-up preparation of pure isomers at the semipreparative level. In practice, a ratio of 99.5:0.5 was not used for semipreparation due to a dramatic drop in separating efficiency of the chiral column over time. It is necessary to rescue resolution efficiency of the column by frequent conditioning with sole isopropanol. The purity of the two separated isomers with cyclohexane/isopropanol or hexane/isopropanol mobile phases at a ratio of 99:1 (*v/v*) was confirmed by HPLC analysis (Figures S2 and S3). The retention times of two peaks eluted with hexane/isopropanol were slightly longer than those eluted with cyclohexane/isopropanol. As alkylpyrazines of low molecular weight (between 94 and 166 with 5−10 carbons) are very volatile, the condensation of organic solvent extracts may cause complete loss of alkylpyrazines. In contrast to the conventional preparation of pure samples for NMR analysis and bioassay, we selected cyclohexane to prepare a concentrated sample for NMR analysis, and hexane to prepare a concentrated sample for bioassay.

### 2.2. Gas Chromatography–Mass Spectrometry

**Figure 4:**
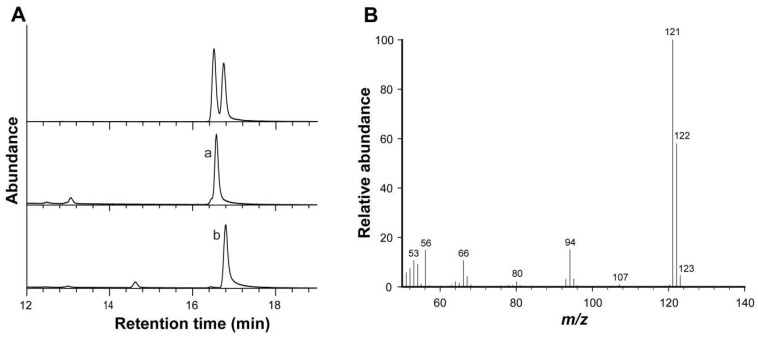
Gas chromatography–mass spectrometry (GC-MS) analysis of EMP using a DB-WAX column. (**A**) GC traces of HPLC-purified EMPa (middle panel) and EMPb (bottom panel) in comparison with the mixture (top panel); (**B**) mass spectra.

To determine the chemical structures of the two regio-isomers in the commercial product, the two HPLC-purified fractions prepared through cyclohexane/isopropanol mobile phase, EMPa and EMPb, were first subjected to GC-MS analysis. The DB-WAX column barely resolved the mixture, EMP (Figure 4A). The two GC peaks had identical mass spectra with dominant ions at *m*/*z* 121 (Figure 4B). As quantitative and qualitative analyses of pyrazines are usually achieved by GC and/or GC-MS, the two GC peaks can be readily identified by comparing elution profile (peak shape and elution sequence) with that present in current study based on following structural elucidation.

### 2.3. NMR

**Figure 5:**
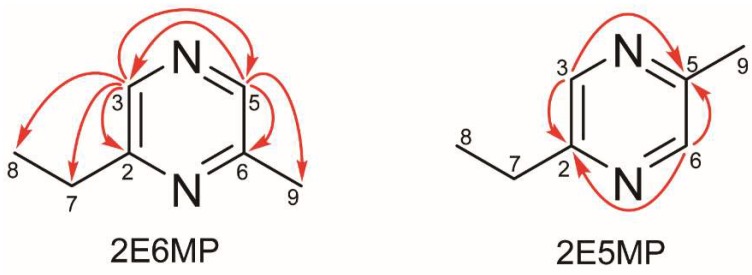
Selected HMBC correlations of EMPa and EMPb.

The two fractions obtained from preparation through cyclohexane/isopropanol mobile phase, EMPa and EMPb, were further concentrated to confirm their chemical identity by using 2D-HSQC- and -HMQC-NMR technique. The peak assignments are shown in Table 1. The apparent C-H correlations evidenced by HSQC spectra helped assignments of C7, C8 and C9 (Figures S4A and S5A). The structures of EMPa and EMPb were characterized by analysis of the HMBC data (Figures S4B and S5B). The signal at *𝛿*_H_ 8.29 showed long-range correlations with two methine signals at *𝛿*_C_ 140.7 and 141.7 of pyrazine ring in EMPa. On the contrary, long-range correlations between the signals at *𝛿*_H_ 8.39, 8.41 and the two methine signals at *𝛿*_C_ 142.7 and 143.5 of pyrazine ring were completely absent in EMPb. Therefore, the presence and absence of methine C–H correlation on pyrazine ring clearly indicated that the structures of EMPa and EMPb were 2E6MP and 2E5MP, respectively (Figure 5). NMR spectroscopy is widely used in determining intramolecular and intermolecular interactions, as well as analyzing structural features. Our study presented an excellent example that used ^1^H-NMR and ^13^C-NMR with 2D-HMBC technique to verify the separation of two closely related regio-isomers in the commercial product, EMP with HPLC. The results of this study can also provide guidance to the rapid identification of 2E6MP and 2E5MP in food samples with coupled HPLC-NMR technique. Our result is in agreement with published elution order of 2E6MP and 2E5MP in GC-MS analysis with DB-WAX, DB-5 and HP-5ms columns [5,36,37,38,39].

### 2.4. HPLC Quantitation

Based on above established HPLC elution order of 2E6MP and 2E5MP, the two fractions obtained from preparation through hexane/isopropanol mobile phase, EMPc and EMPd, were characterized as 2E6MP and 2E5MP, respectively. The external standard method was used to obtain a regression equation, A = 407.2587C + 54.0169, where A is the peak area and C is the concentration of the standard compound (µg/μL). The standard compound showed excellent linearity (R^2^ = 0.9999, *P* < 0.0001) in selected concentration ranges. With the obtained calibration curve, the concentrations of 2-mL preparative EMPc, EMPd solutions were determined to be 10.26 and 8.31 µg/µL, respectively.

### 2.5. Bioassay

**Figure 6:**
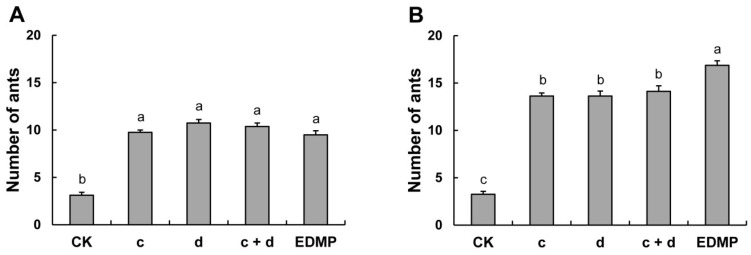
Alarm response of *S. invicta* workers to HPLC-purified EMPc, EMPd, and their mixture. (**A**) Dose = 10 ng; (**B**) Dose = 100 ng. **c** = EMPc, **d** = EMPd. Means for the same dose having no letter in common on each bar of the histogram (a, b, and c) are significantly different (*P* < 0.05, Tukey-HSD test).

HPLC-purified isomers were tested for EAG responses in *S. invicta* workers at doses of 10 and 100 µg. The two isomers, EMPc and EMPd, elicited a similar EAG response at the same doses (Table 2), which was not significantly different from the response triggered by the same dose of mixed isomers (EMPc + EMPd, 1:1), suggesting that there exists a sole addition effect between the two isomers. Furthermore, the EAG response to the real alarm pheromone, EDMP was slightly greater, but not significantly, than that to all EMP treatments at both doses (*d*.*f*. = 3.36, *F* = 0.54, *P* = 0.6604 for dose of 10 µg; *d*.*f*. = 3.36, *F* = 1.26, *P* = 0.3032 for dose of 100 µg). The EAG results were confirmed by the alarm behavior bioassays with the two HPLC-purified isomers at doses of 10 and 100 ng. The data from the behavioral bioassays showed that the number of workers responding to odors was significantly more than the number of workers responding to hexane (Figure 6). At a dose of 10 ng, no significant difference was recorded in the number of workers among the four odor treatments. At a dose of 100 ng, the number of workers responding to EDMP was significantly more than that responding to EMP isomers and their mixture. However, the two isomers elicited equal alarm response, and the activity of the mixture of both isomers (1:1) was similar to that of individual isomer. Almost no difference in physicochemical properties of the two regio-isomers of EMP may account for the lack of bioactivity differences in both EAG and alarm behavioral responses [40].

In both EAG and alarm behavior experiments, EMP was proved as active, but with different activation thresholds (possible 1000-time difference). Similar patterns of EAG and alarm behavior responses have been observed testing fire ant alarm pheromone EDMP and other analogues in previous studies [25,26]. Taken together, these results confirm the identification of fire ant alarm pheromone component, EDMP [24].

Recently, the role of a commercially available mixture of fire ant alarm pheromone component, 2-ethyl-3,5(6)-dimethylpyrazine in mediating fire ant-phorid fly interactions has been investigated. A parasitoid of *S. invicta*, *Pseudacteon tricuspis* was attracted to alarm pheromone isomer and six other structurally related alkylpyrazine analogues [41]. Furthermore, among the fire ant alarm pheromone and six other closely related alkylpyrazine analogues, 2,3-diethyl-5-methylpyrazine and a mixture (possibly 2-ethyl-3(5 or 6)-methylpyrazine) consistently elicited both EAG and behavior attraction to *Pseudacteon* flies [42]. The significant alarm activities of EMP in this study along with the other three analogues, 2-ethyl-3,5-dimethylpyrazine, 2,3,5-trimethylpyrazine, and 2,3-diethyl-5-methylpyrazine, in Guan et al. [26] are consistent with reports on the activities of alkylpyrazine analogues in phorid fly attraction [41,42]. It is plausible that some of these active alkylpyrazine analogues could be minor components of fire ant alarm pheromones.

Several studies have confirmed the nutty odor of 2E6MP and 2E5MP in food samples. 2E6MP had the highest odor intensity among the alkylpyrazines found in wild rice, which appeared to contribute to the unique ‘almond’ aroma of wild rice [38]. 2E5MP was confirmed to contribute to roasty odor of tartary buckwheat tea [43]. As 2E5MP was not well resolved from 2E6MP in GC-olfactory analysis on an HP-5ms column, it was possible that both 2E6MP and 2E5MP contributed to the reported aroma. 2E6MP was characterized as a ‘flowery’ and ‘fruity’ odor in the coffee brew aroma [44]. Both 2E6MP and 2E5MP were considered to be principal contributors of sesame seed oil flavor [36]. Ants are generally attracted to emission sources of alarm pheromones that can also function as recruitment stimuli to recruit large numbers of nestmates to a pheromonal point source with incredible rapidity [45,46]. Therefore, 2E6MP and 2E5MP present in food products may act as attractants to ant foragers, resulting in quicker location of food sources. Ants may have evolved through natural selection to utilize these alkylpyrazines present in food products to improve foraging effectiveness and colony fitness. This characteristic behavior to alarm substances has led to the development of new strategies in ant control. When incorporated into baits, 4-methyl-3-heptanone, alarm pheromonal component from leaf-cutting ants (*Atta* spp.), has previously proved to substantially increase the attractiveness and harvest of the treated bait and the discovery of nearby untreated bait in the field [47,48]. Fire ant workers exhibit rapid alarm response to alarm pheromone component and its analogues [25,26]. These active alkylpyrazine analogues stimulated the same behaviors as alarm pheromone when incorporated in food baits and could be used in formulating effective attractants to improve harvest of toxic bait by fire ants. Future work will test the effectiveness of alkylpyrazines on bait harvest and substantial control of the imported fire ants in the field.

## 3. Materials and Methods

### 3.1. Chemicals and Solvents 

HPLC grade cyclohexane, hexane and isopropanol purchased from CNW Technologies GmbH (Düsseldorf, Germany) were used without further purification. EMP (2-ethyl-5(6)-methylpyrazine, 98%) and MEOP (2-methyl-3(5 or 6)-ethoxypyrazine, 99%) were obtained from Sigma-Aldrich (St. Louis, MO, USA). 2-Ethyl-3-methylpyrazine (99%) and deuterated chloroform were purchased from Acros Organics (Geel, Belgium). EDMP (2-ethyl-3,6-dimethylpyrazine) was synthesized as described in Hu et al. [49].

### 3.2. HPLC

HPLC analysis of EMP was carried out on an Agilent HP 1260 instrument (Agilent Technologies, Palo Alto, CA, USA) equipped with a UV detector. Separation was accomplished on a Chiralpak AD-H (amylose tris-(3,5-dimethylphenyl-carbamate) column (250 mm × 4.6 mm i.d., 5 µm particle size), obtained from Daicel Chemical Industries (Tokyo, Japan). A solution of EMP at 10 µg/µL was prepared with cyclohexane or hexane for both analytical separation and semipreparation. All chromatographic experiments were performed in the isocratic mode. The mobile phase was either cyclohexane/or hexane/isopropanol at a flow-rate of 1 mL/min. The effluent was monitored at a wavelength of 278 nm. Various percentages (0.5–10%) of isopropanol were tested to optimize the separation efficiency of the mobile phase. The injection volume was 1 µL in analytical separations. In semipreparative separation, the injection volume was 5 µL for both cyclohexane/isopropanol (99:1) and hexane/isopropanol (99:1) eluents. Each peak fraction was manually collected according to the elution profile. After two-week semipreparative separation, the collected fractions were combined. The two pooled fractions prepared through cyclohexane/isopropanol system, designated as EMPa and EMPb, were concentrated under a gentle stream of nitrogen to 0.3 mL, to which 0.2 mL of CDCl_3_ was added for NMR analyses. The other two pooled fractions prepared through hexane/isopropanol system, designated as EMPc and EMPd, were concentrated to 2 mL in the same manner and subjected to HPLC quantitation.

### 3.3. Gas Chromatography–Mass Spectrometry

Electron impact (EI) capillary GC-MS analysis of commercial EMP, HPLC-purified EMPa and EMPb was carried out using an Agilent 7890A GC coupled to a 5975C mass selective detector. A polar capillary column, DB-WAX (30 m × 0.25 mm i.d., 0.25 μm particle size) (Agilent Technologies, USA), was used to test for elution order of the two EMP isomers. The GC oven temperature was programmed from 90 °C (isothermal for 2 min) to 240 °C at 15 °C /min and held for 10 min. The injection temperature was set at 230 °C, and the transfer line temperature was set at 250 °C. The 70 eV EI spectra of EMP was confirmed by the NIST GC-MS library. 

### 3.4. NMR Spectroscopy

One-dimensional and 2D-NMR spectra of HPLC-purified EMPa and EMPb were measured on an Agilent DD2 500 MHz spectrometer (Agilent Technologies, Santa Clara, CA, USA) in CDCl_3_ with tetramethylsilane (TMS) as the internal standard. An Agilent standard two-pulse sequence with presaturation option was used to suppress strong CH_2_ signal of cyclohexane.

### 3.5. HPLC Quantitation

To quantitate 2E6MP and 2E5MP in EMPc or EMPd fraction, 2-ethyl-3-methylpyrazine (Figure 1) was used as the external standard. A stock solution of standard compound 2-ethyl-3-methylpyrazine (50 µg/µL) was prepared and then diluted to a series of concentrations ranging from 1 to 32 µg/μL. All dilutions were transferred to the HPLC autosampler, and 1 μL of each dilution was used for HPLC analysis under the same conditions as above with a hexane/isopropanol mobile phase system. A standard curve was calculated by linear regression analysis. The concentration of pyrazine in the 2-mL hexane fraction was calculated against the standard curve. The hexane fractions of EMPc and EMPd were, thereafter, diluted (or concentrated) to 10, 1 µg/µL for EAG study, and to 10, 1 ng/µL for behavioral study. A mixture of equal volume of both EMPc and EMPd solutions at the same concentration was bioassayed to determine the synergistic effect.

### 3.6. Insects

We collected eight mature colonies of *S. invicta* on the campus of South China Agricultural University (Guangzhou, Guangdong Province, China). Each colony contained workers, broods, and queens and was transferred in plastic jars coated with Fluon to prevent escape. All colonies were reared in the laboratory and fed with 10% honey solution, and larvae of *Tenebrio molitor* L.

### 3.7. Bioassays

The EAG sensitivity of *S. invicta* workers was determined by conventional EAG methods using a commercially available IDAC-4 system (Syntech, Kirchzarten, Germany). Details of the method have been described elsewhere [25,26,50]. Isolated ant heads with antennae were used for the EAG test. Each antennal preparation (*n* = 10 for each compound) was challenged in the following order: Hexane control, EDMP, EMPc or EMPd, EMPc + EMPd; hexane control. For analyses, EAG response to the solvent control was deducted from the EAG amplitudes elicited by the test compounds. Means of corrected EAG data across all test compounds at same dose were compared by Tukey-Kramer HSD comparison test (*P* < 0.05) [51].

Behavioral bioassays were conducted by the same method as described in previous reports [25,26]. The response to a test compound was evaluated on the number of workers that were running out of the quiescent ant group within 30 s. A test series of EDMP, EMPc, EMPd, EMPc + EMPd of the same dose (10, or 100 ng) was replicated eight times. Bioassay data were determined to be normally distributed and then analyzed using one-way analysis of variance (ANOVA) followed by Tukey-Kramer HSD comparison test (*P* < 0.05) to establish significant differences among the treatments [51].

## 4. Conclusions

In this study, two regio-isomers of EMP were effectively separated by using a Chiralpak AD-H column with two mobile phases: Cyclohexane/isopropanol, and hexane/isopropanol. The GC-MS data were used to ensure the elution order of the two regio-isomers, 2E6MP and 2E5MP, on a DB-WAX column. Via 1D-^1^H, ^13^C- and 2D-HSQC, -HMBC-NMR, a complete assignment of all proton signals within the spectra was performed. The 2D-NMR-spectroscopic characterization, and electrophysiological and alarm activities of 2E6MP and 2E5MP were reported here for the first time. 2D-HMBC-NMR showed that there existed apparent presence of long-range correlations with two methine signals of pyrazine ring in 2E6MP. Based on 2D-NMR spectroscopic data, 2E6MP was found to be eluted first followed by 2E5MP on the Chiralpak AD-H column. The elution order of the two regio-isomers in GC-MS analysis on a DB-WAX column was the same as that in HPLC analysis. The two regio-isomers proved to be equally active in both EAG and alarm behavior bioassay with *S. invicta* workers. This strategy can also be adapted to separate oxygen-containing pyrazine, 2-methyl-3(5 or 6)-ethoxypyrazine.

This work demonstrates that chiral column can be used for separation of closely related non-chiral regio-isomers, and cyclohexane/isopropanol as a mobile phase is capable of resolving volatile pyrazines with very low molecular weight for subsequent NMR study. 2D-NMR analyses of preparative fractions allowed further structural characterization and differentiation of the two regio-isomers. The strategy presented here provides time efficiency because an HPLC run on the chiral column took only a few minutes and NMR analysis was performed directly on the preparative fractions.

## Figures and Tables

**Table 1:** ^1^H- and ^13^C-NMR data of 2-ethyl-5(6)-methylpyrazine a (EMPa) and 2-ethyl-5(6)-methylpyrazine b (EMPb) ^a^.

EMPa			EMPb		
No.	𝛿_H_ (mult, *J*, Hz)	𝛿_C_	No.	𝛿_H_ (mult, *J*, Hz)	𝛿_C_
2		157.7	2		155.4
3	8.29 (s)	140.7	3	8.39 (s)	142.7
5	8.29 (s)	141.7	5		150.7
6		152.8	6	8.41 (s)	143.5
7	2.81 (q, 7.62)	28.8	7	2.86 (q, 7.60)	28.1
8	1.34 (t, 7.62)	13.6	8	1.39 (m)	13.4
9	2.56 (s)	21.4	9	2.59 (s)	20.8

^a^ Recorded in CDCl_3_ at 500 MHz for ^1^H-NMR and 125 MHz for ^13^C-NMR.

**Table 2:** Electroantennogram (EAG) response of *S. invicta* workers to HPLC-purified EMPc, EMPd, and their mixture.

Compound	EAG (mV ± SE)	
	Dose = 10 µg	Dose = 100 µg
EMPc	0.47 ± 0.056	1.43 ± 0.110
EMPd	0.54 ± 0.078	1.49 ± 0.112
EMPc + EMPd	0.50 ± 0.047	1.41 ± 0.100
EDMP	0.57 ± 0.050	1.66 ± 0.073

## Supplementary Materials

The supplementary materials are available online. Figure S1. HPLC chromatogram of 2-methyl-3(5 or 6)-ethoxypyrazine. Mobile phase: Hexane-isopropanol 99.5:0.5. Figure S2. HPLC chromatograms of HPLC-purified EMPa (middle panel) and EMPb (bottom panel) in comparison with the mixture (top panel). Figure S3. HPLC chromatograms of HPLC-purified EMPc (middle panel) and EMPd (bottom panel) in comparison with the mixture (top panel). Figure S4. HSQC-and HMBC-2D NMR spectra of EMPa, 2E6MP. A: HSQC; B: HMBC. Figure S5. HSQC-and HMBC-2D NMR spectra of EMPb, 2E5MP. A: HSQC; B: HMBC.
