# Supplementary material for: HPLC Separation of 2-Ethyl-5(6)-methylpyrazine and Its Electroantennogram and Alarm Activities on Fire Ants (Solenopsis invicta Buren)
Source: Molecules. 2018 Jul 7;23(7):1661. doi: 10.3390/molecules23071661 (PMC6100352; doi:10.3390/molecules23071661)
Supplement: Supplementary file 1 [file molecules-23-01661-s001.pdf]

HPLC Separation of 2-Ethyl-5(6)-methylpyrazine and Its Electroantennogram and Alarm Activities on Fire Ant (*Solenopsis invicta* Buren)

Ya-Ya Li[1,2], Yong-Yue Lu[3], Min Lu[1], Hong-Yi Wei[2,*], Li Chen[1,*]

Supplementary Materials:

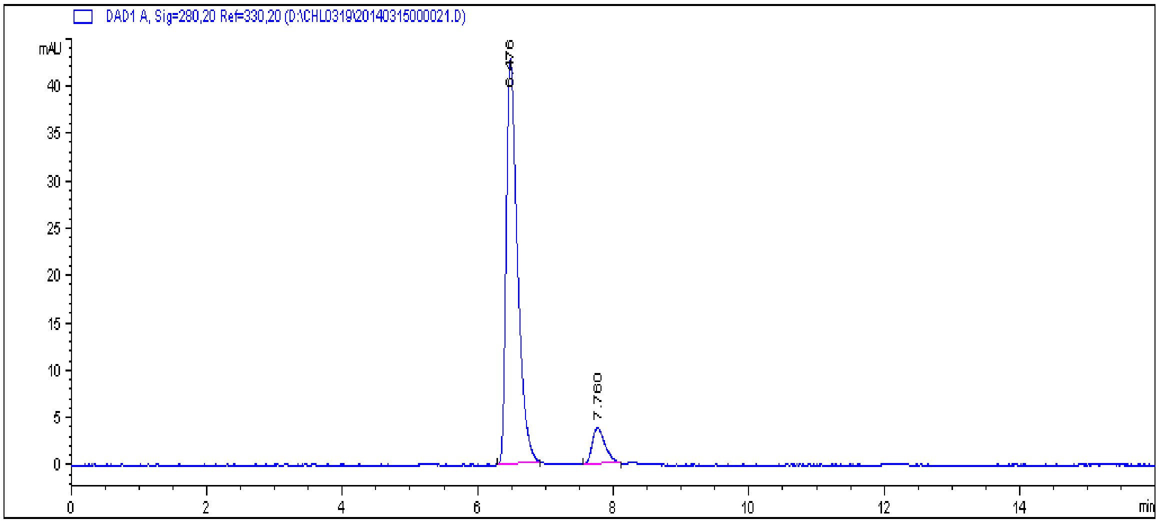

Figure S1. HPLC chromataogram of 2-methyl-3(5 or 6)-ethoxypyrazine. Mobile phase: hexane-isopropanol 99.5:0.5.

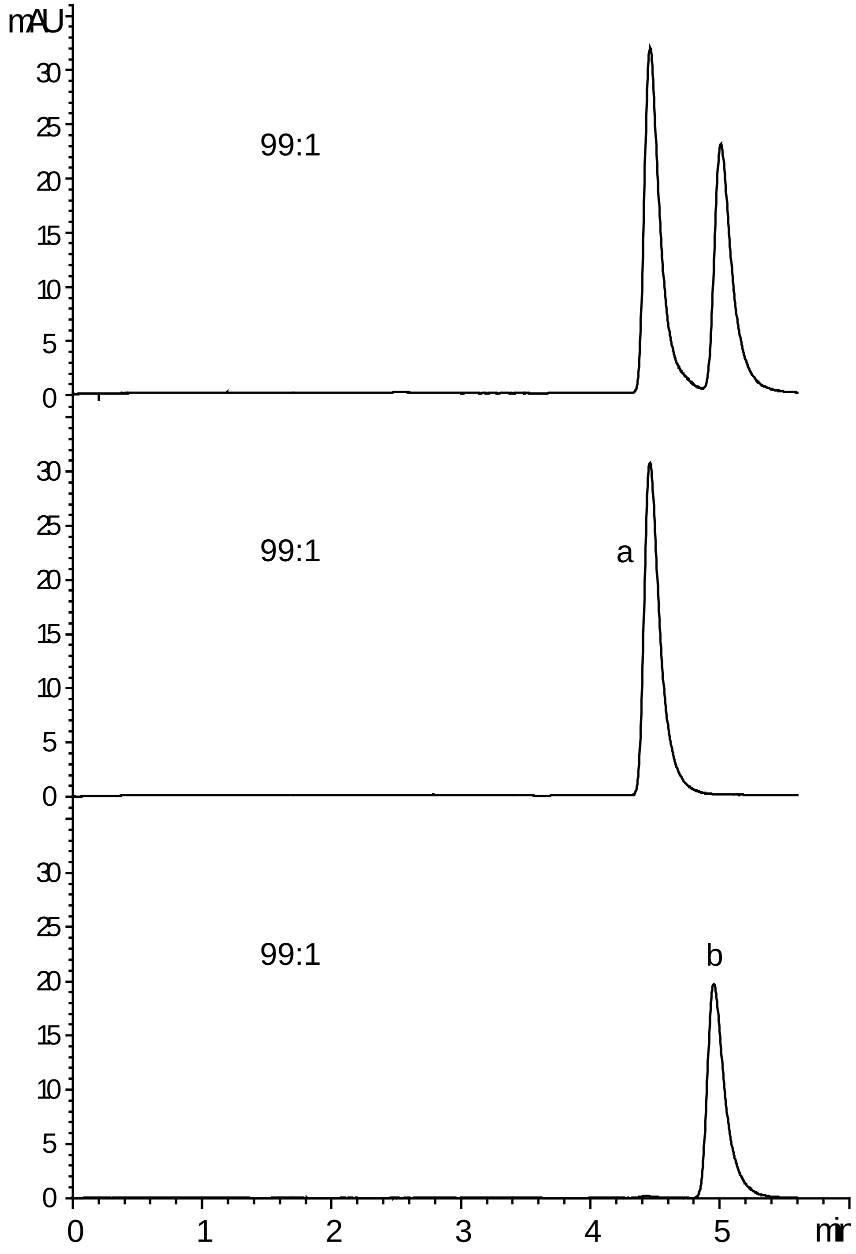

Figure S2. HPLC chromatograms of HPLC−purified EMPa (middle panel) and EMPb (bottom panel) in comparison with the mixture (top panel).

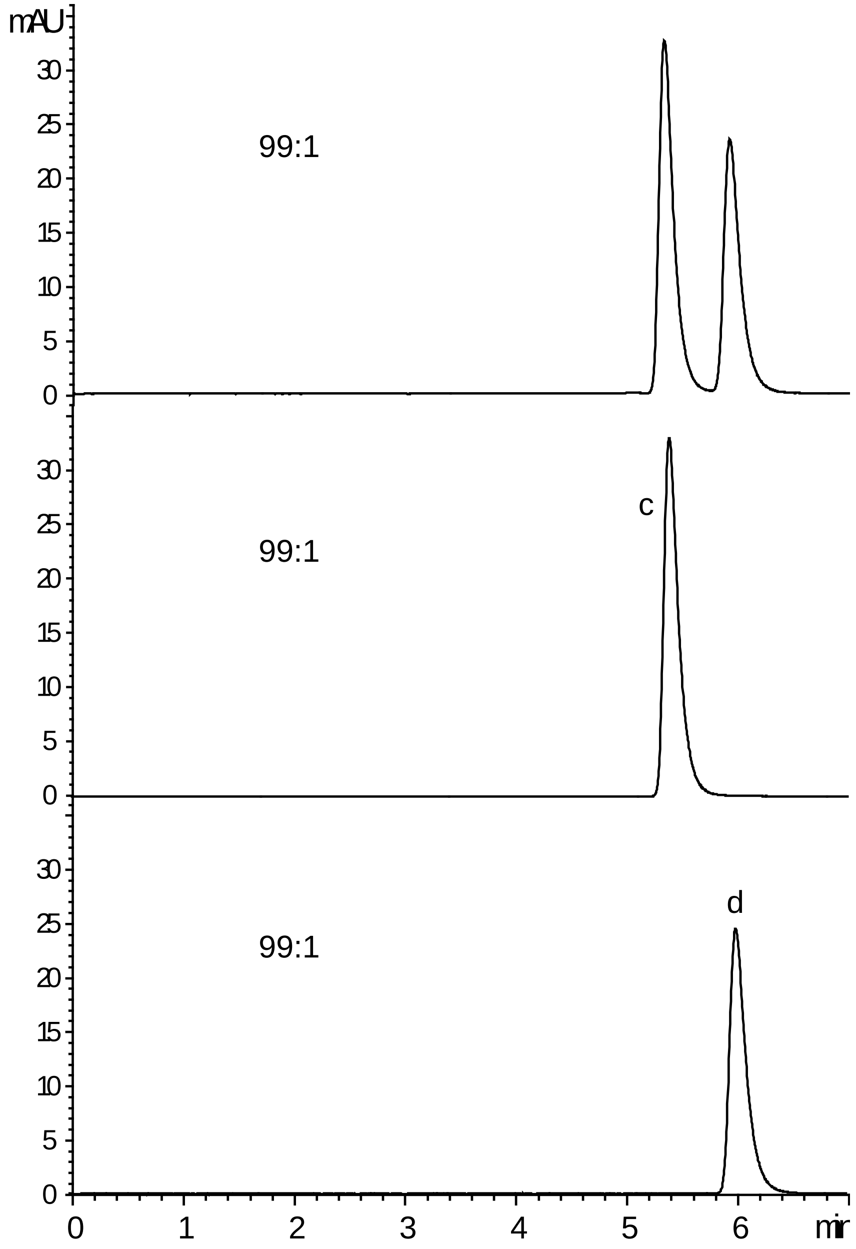

Figure S3. HPLC chromatograms of HPLC−purified EMPc (middle panel) and EMPd (bottom panel) in comparison with the mixture (top panel).

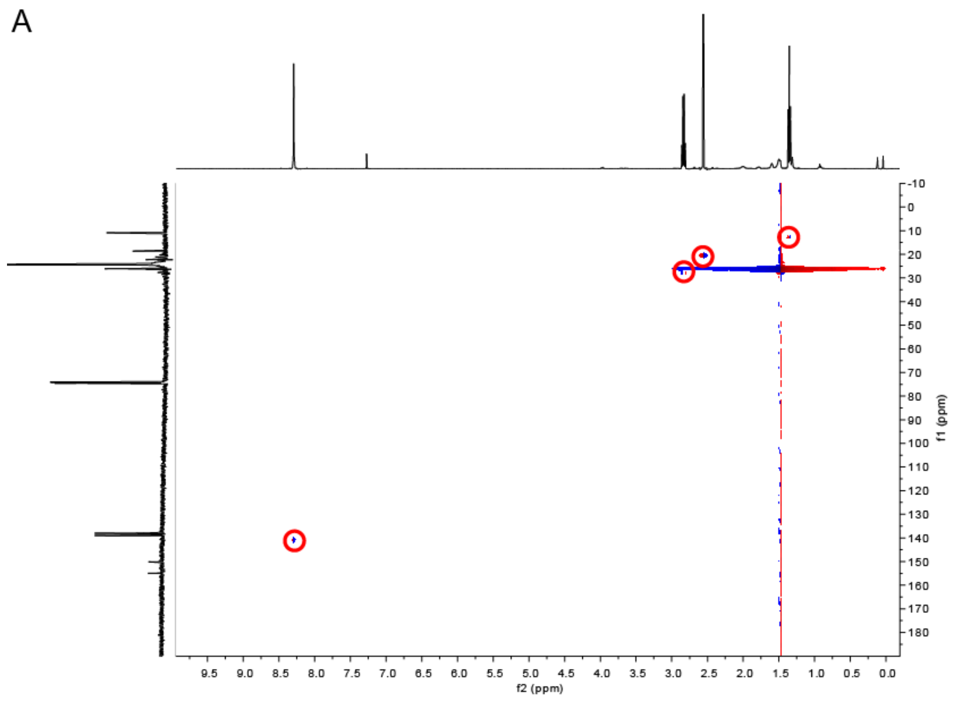

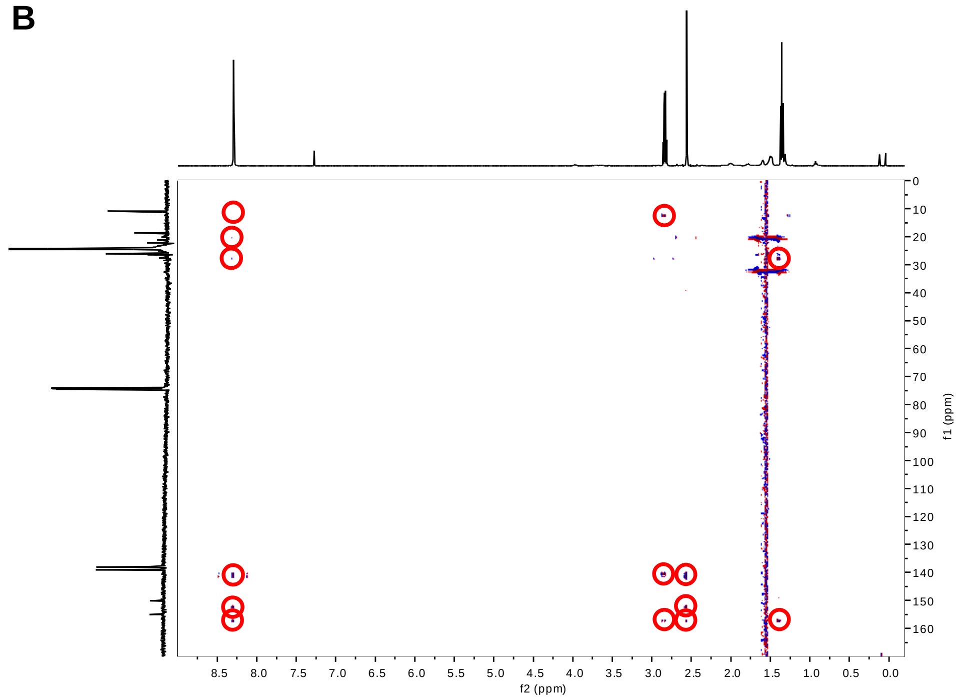

Figure S4. HSQC− and HMBC−2D NMR spectra of EMPa, 2-ethyl-6-methylpyrazine.

A: HSQC; **B**: HMBC.

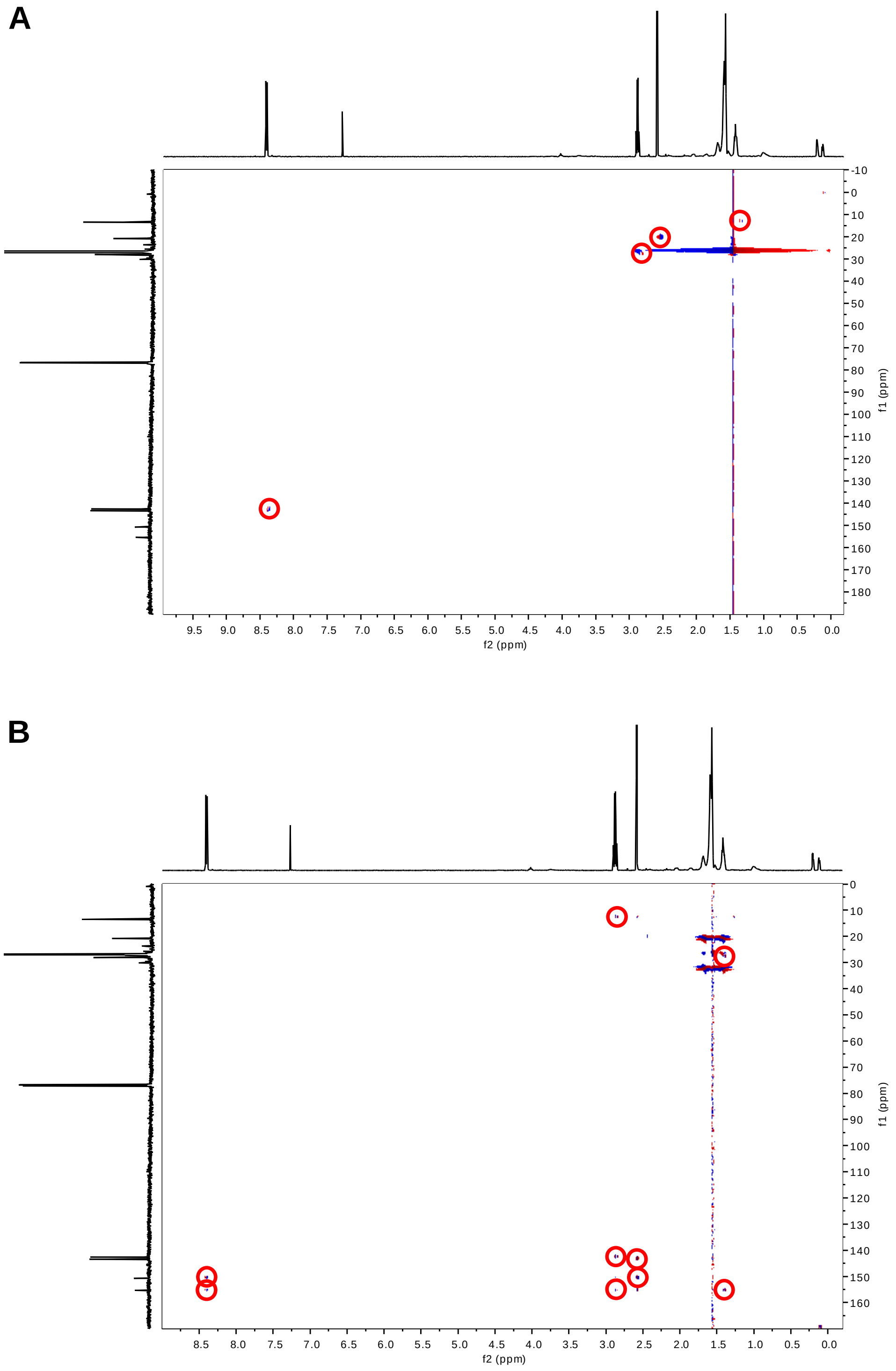

Figure S5. HSQC− and HMBC−2D NMR spectra of EMPb, 2-ethyl-5-methylpyrazine. **A**: HSQC; **B**: HMBC.
